# Ecological change of the gut microbiota during pregnancy and progression to dyslipidemia

**DOI:** 10.1038/s41522-023-00383-7

**Published:** 2023-04-03

**Authors:** Xu Yang, Mingzhi Zhang, Yuqing Zhang, Hongcheng Wei, Quanquan Guan, Chao Dong, Siting Deng, Hein Min Tun, Yankai Xia

**Affiliations:** 1 State Key Laboratory of Reproductive Medicine, School of Public Health, Nanjing Medical University, Nanjing, China; 2 Key Laboratory of Modern Toxicology of Ministry of Education, School of Public Health, Nanjing Medical University, Nanjing, China; 3 Women’s Hospital of Nanjing Medical University, Nanjing Maternity and Child Health Care Hospital, Nanjing, China; 4 The Jockey Club School of Public Health and Primary Care, Faculty of Medicine, The Chinese University of Hong Kong, Hong Kong SAR, China; 5 Li Ka Shing Institute of Health Sciences, The Chinese University of Hong Kong, Hong Kong SAR, China; 6 HKU-Pasteur Research Pole, School of Public Health, Li Ka Shing Faculty of Medicine, The University of Hong Kong, Hong Kong SAR, China

**Keywords:** Health care, Metagenomics

## Abstract

The composition of the gut microbiome was previously found to be associated with clinical responses to dyslipidemia, but there is limited consensus on the dynamic change of the gut microbiota during pregnancy and the specific microbiome characteristics linked to dyslipidemia in pregnant women. We collected fecal samples from 513 pregnant women at multiple time points during pregnancy in a prospective cohort. Taxonomic composition and functional annotations were determined by 16S rRNA amplicon sequencing and shotgun metagenomic sequencing. The predictive potential of gut microbiota on the risk of dyslipidemia was determined. The gut microbiome underwent dynamic changes during pregnancy, with significantly lower alpha diversity observed in dyslipidemic patients compared to their healthy counterparts. Several genera, including *Bacteroides*, *Paraprevotella*, *Alistipes*, *Christensenellaceae R7 group*, *Clostridia UCG-014*, and *UCG-002* were negatively associated with lipid profiles and dyslipidemia. Further metagenomic analysis recognized a common set of pathways involved in gastrointestinal inflammation, where disease-specific microbes played an important role. Machine learning analysis confirmed the link between the microbiome and its progression to dyslipidemia, with a micro-averaged AUC of 0.824 (95% CI: 0.782-0.855) combined with blood biochemical data. Overall, the human gut microbiome, including *Alistipes* and *Bacteroides*, was associated with the lipid profile and maternal dyslipidemia during pregnancy by perturbing inflammatory functional pathways. Gut microbiota combined with blood biochemical data at the mid-pregnancy stage could predict the risk of dyslipidemia in late pregnancy. Therefore, the gut microbiota may represent a potential noninvasive diagnostic and therapeutic strategy for preventing dyslipidemia in pregnancy.

## Introduction

Dyslipidemia is one of the most common metabolic disorders characterized by abnormal lipid levels^1^, with the prevalence estimated at 53.0% in North America^2^ and as high as 34.0% in China^3^. Between 2010 and 2030, elevated blood cholesterol (CHOL) levels in the population will lead to an increase of approximately 9.2 million cardiovascular events^4^. Pregnancy induces metabolic and immunological changes resulting in elevated triglyceride (TG) and total cholesterol levels to meet the needs of fetal growth^5^. In a normal pregnancy, women show a 30–50% physiological increase in plasma CHOL and TG concentration, with a 2–4 fold increase in the third trimester^6,7^. Dyslipidemia during pregnancy increases the risk of pregnancy complications, such as gestational diabetes mellitus (GDM), preterm birth, and cardiovascular risk later in life^8^.

The intestinal microbiota is essential in maintaining the host’s metabolic function and lipid metabolism balance^9^. Epidemiological studies revealed that patients with dyslipidemia were characterized by a low richness of the gut microbiota community^10^, and specific gut microbiota taxa showed significant associations with lipid levels^9^. In addition, studies using washed microbiota transplantation treatment in dyslipidemia patients have revealed the gut microbiome’s role in the treatment of dyslipidemia^11^. These studies provided evidence for the associations of blood lipid levels with the gut microbiota and highlighted the gut microbiome as a potential therapeutic target. However, it is unclear whether blood lipid levels during pregnancy are associated with gut microbiota. Evidence indicated that the community structure of the gut microbiota changes dramatically throughout pregnancy^12,13^, and perturbed gut microbiota during pregnancy was found to be associated with multiple commodities such as GDM^14^ and inflammatory bowel disease^15^. Evidence also indicated the role of gut microbiota in predicting metabolic disorders^16,17^. However, whether the dynamic change of gut microbiota during gestation was associated with dyslipidemia and whether the gut microbiota structure can predict the risk of dyslipidemia is unknown.

In the current study, we investigated the dynamic changes in gut microbiota compositions during pregnancy and the associations of gut microbiota profiles with maternal lipid profile and dyslipidemia, aiming to determine the potential role of the gut microbiome in the pathophysiology of dyslipidemia during pregnancy. In addition, we explored whether gut microbiota in mid-pregnancy can predict the risk of dyslipidemia in late pregnancy, thus aiming to offer potential noninvasive diagnostic and therapeutic strategies for dyslipidemia during pregnancy.

## Results

### Clinical characteristics of the study population

**Fig. 1:**
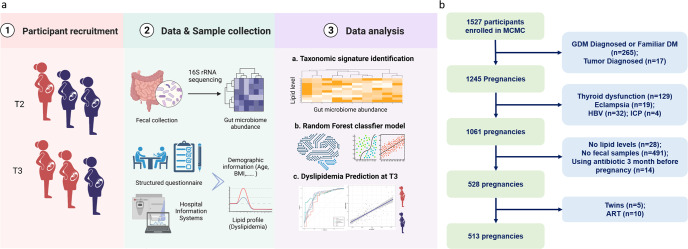
Study design and flow diagram of the population. **a** Study design (Created with BioRender.com). **b** Flow diagram of the population included in this study.

**Fig. 2:**
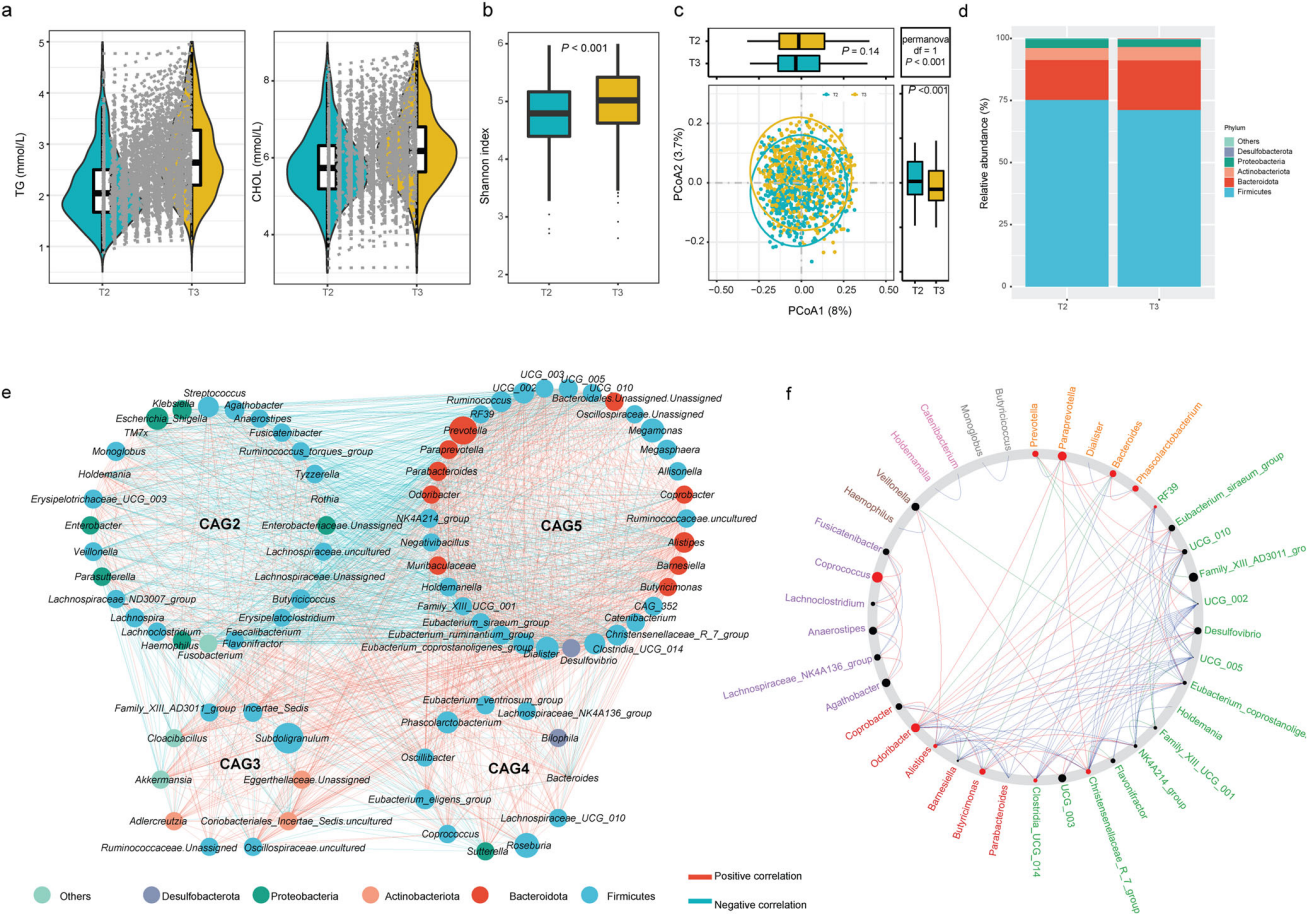
Dynamic change of lipid levels and gut microbiota structure during pregnancy. **a** Violin plots showing the TG and CHOL levels during pregnancy. The dotted lines indicated the trend of change within the participant. **b** Boxplots showing the change of alpha diversity indexes during pregnancy. The center line denoted the median. The boxes covered the 25th and 75th percentiles, and the whiskers extended to the most extreme data point, which was no more than 1.5 times the length of the box away from the box. Points outside the whiskers represented outlier samples. *P* values in the plots showed *P* obtained from the Wilcoxon rank-sum test. **c** Change of beta diversity indexes during pregnancy. The center line denoted the median. The boxes covered the 25th and 75th percentiles, and the whiskers extended to the most extreme data point, which was no more than 1.5 times the length of the box away from the box. Points outside the whiskers represented outlier samples. *P* values in the boxplots and blank squares showed *P* obtained from the Wilcoxon rank-sum test and PERMANOVA test, separately. **d** Comparison of relative abundance of phylum. **e** Network plots of genera in participants during pregnancy. Each node represented a genus. The size of each node correlates to the mean abundance of each genus across all samples. Nodes were shown if the abundance of the respective genus was significantly correlated with each other (FDR < 0.05). Red line: positive correlation; Blue line: negative correlation. Each node was colored according to its phylum. **f** Most common sub-network between the T2 and T3 network. All nodes belonging to the same community were randomly assigned a same color. Grayed-out nodes represented the ones present in both but directly interacted with the common sub-network in either T2 or T3. Node sizes were proportional to their scaled NESH score, and a node was colored red if its betweenness increased from T2 to T3. Hence, big and red nodes were particularly important ‘drivers’.

The study was based on the Mother and Child Microbiome Cohort (MCMC) Study^16,18^. The study design is presented in Fig. 1a. Pregnant women were recruited from the affiliated hospital of Nanjing Medical University between 2017 and 2018. A total of 1527 pregnant women were enrolled in the longitudinal study. Stool samples were collected in the second (T2, 24.14 ± 0.95 weeks) and third (T3, 32.11 ± 0.59 weeks) trimesters of pregnancy. Negative control samples (*n* = 10) were also included during fecal samples’ collection, transportation, and DNA extraction. All collected samples were stored at −20 °C in the refrigerator until transferred to the laboratory at −80 °C (Fig. 1). The study encompassed a total of 513 individuals with a median age of 29.2 years (Supplementary Tables 1 and 2). About two-thirds of the study population had median education (high school to bachelor’s degree, 73.6%) and were primiparous (74.1%). 6.43% of participants (*n* = 33) were overweight or obese before pregnancy. As shown in Fig. 2a, the lipid levels increased significantly from T2 to T3 (Wilcoxon rank-sum test, *P* < 0.001), with the dashed line indicating the trend per person. Among those women with normal lipid levels at T2, 32.9% developed hyperlipidemia at T3. A small number of participants (2.5% with dyslipidemia at T2) returned to normal lipid levels at T3.

### Change of intra-individual microbiota composition over time during pregnancy

We evaluated dynamic changes in microbiota diversity and composition from T2 to T3. A Principal Coordinates Analysis (PCoA) on the unweighted Unifrac distance was performed on the overall microbiota communities at T2 to T3. Shannon index increased significantly during gestation in all participants (Fig. 2b). PERMANOVA test indicated that the composition of gut microbial communities significantly differed between T2 and T3 (PERMANOVA test, *P* < 0.001, Fig. 2c). In addition, compositional stability changed with a decrease in Firmicutes, and an increase in Bacteroidetes from T2 to T3 (Fig. 2d).

To identify the structural changes in the community, we constructed Co-abundance Groups (CAGs) based on the spearman correlation of genera. Five robust CAGs during pregnancy were identified (Supplementary Fig. 1a). Genera in the same CAG were positively associated with each other (Fig. 2e). Among these, CAG2, CAG3, and CAG5 were significantly enriched at T3 (Wilcoxon rank-sum test, *P* < 0.05; Supplementary Fig. 1b). CAGs depleted in T2 were mainly composed of genera of Firmicutes (Fig. 2e). NetShift was used to identify potentially important “taxonomic drivers” from T2 to T3 and several potential taxonomic drivers involved in the changes of microbial correlations between T2 and T3. The top taxonomic drivers were *Paraprevotella, Prevotella, Odoribacter*, and *Butyricimonas* (Fig. 2f), mainly composed of CAG5. Similarly, we constructed CAGs at both the second and third trimesters. The network structure was significantly different between the T2 and T3 groups (Supplementary Fig. 1c), with various top microbiota taxa and distinct patterns of microbial interactions. These results indicated that gut microbiota changed significantly during pregnancy and that specific gut microbiota taxa could play a vital role in microbial ecological changes during pregnancy.

### Associations of gut microbiota composition with dyslipidemia during pregnancy

**Fig. 3:**
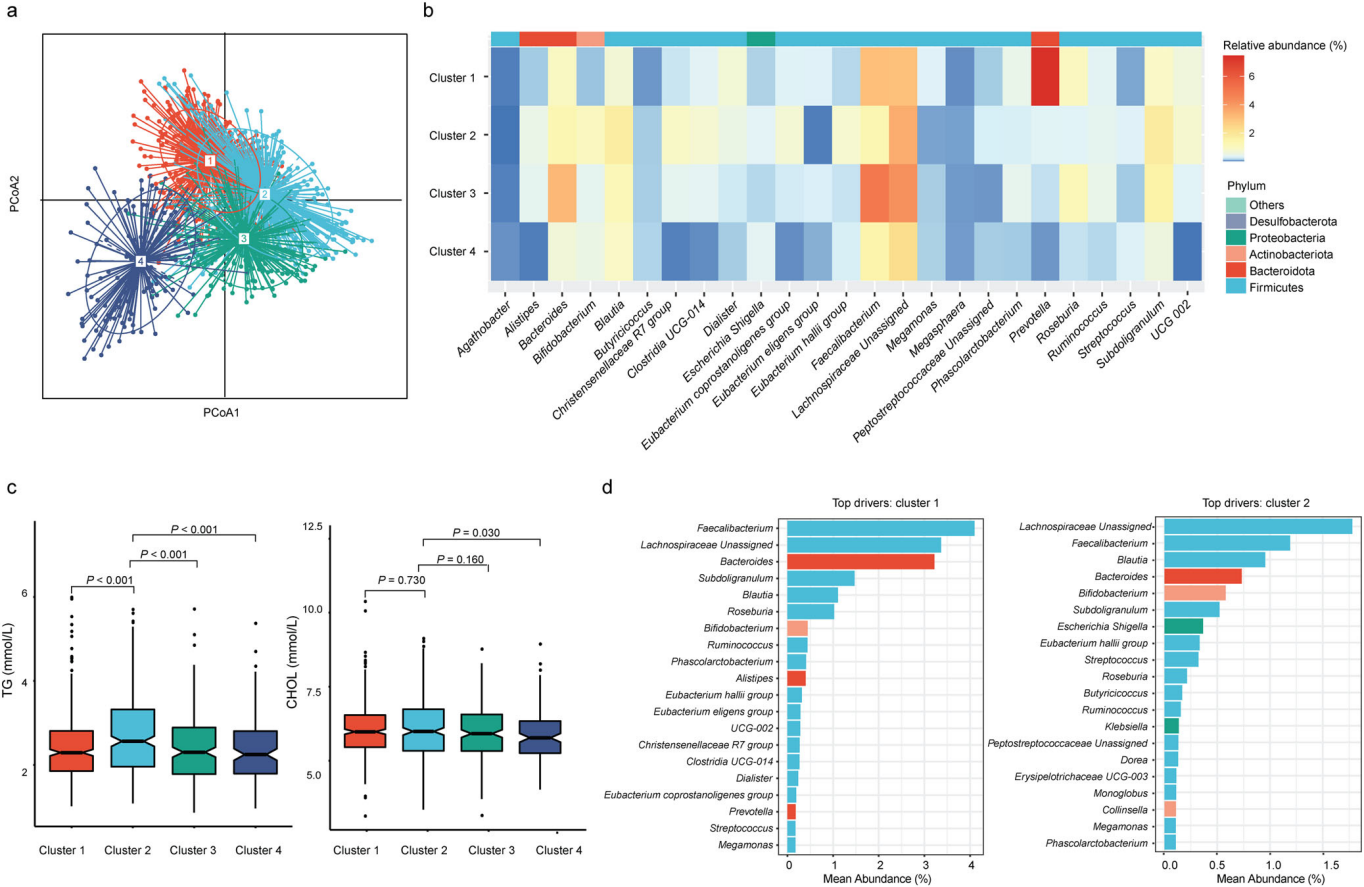
The entire dataset formed four distinct clusters based on the lowest Laplace approximation. **a** Principal coordinate analysis plot based on genera. **b** Heatmap showing the relative abundance of the 25 most dominant bacterial genera per DMM cluster. **c** Boxplots showing the lipid levels per DMM cluster. The center line denoted the median. The boxes covered the 25th and 75th percentiles, and the whiskers extended to the most extreme data point, which was no more than 1.5 times the length of the box away from the box. Points outside the whiskers represented outlier samples. *P* values in the plots showed *P* obtained from the Wilcoxon rank-sum test. **d** Barplots showing the relative abundance of genera in DMM cluster 1 and cluster 2. Each bar was colored according to its phylum.

To determine whether microbiota community structure was associated with lipid levels during pregnancy, we used the Dirichlet multinomial mixtures (DMM) model to explore specific clusters based on gut microbial community structure. Four DMM clusters were identified based on the lowest Laplace approximation score (Fig. 3a; Supplementary Fig. 2a). Dominant bacterial genera (top 25) differed among DMM clusters, and gut community structure changed dramatically during gestation (Fig. 3b). Participants in DMM cluster 2 showed higher TG and CHOL levels compared to other clusters (Fig. 3c), where the dominant genera included *Faecalibacterium, Blautia*, and *Bacteroides* (Fig. 3d). The dominant genera in the other two clusters (Cluster 3 and Cluster 4) were mainly composed of *Lachnoclostridium.Unassigned* and *Prevotella*, separately (Supplementary Fig. 2b).

**Fig. 4:**
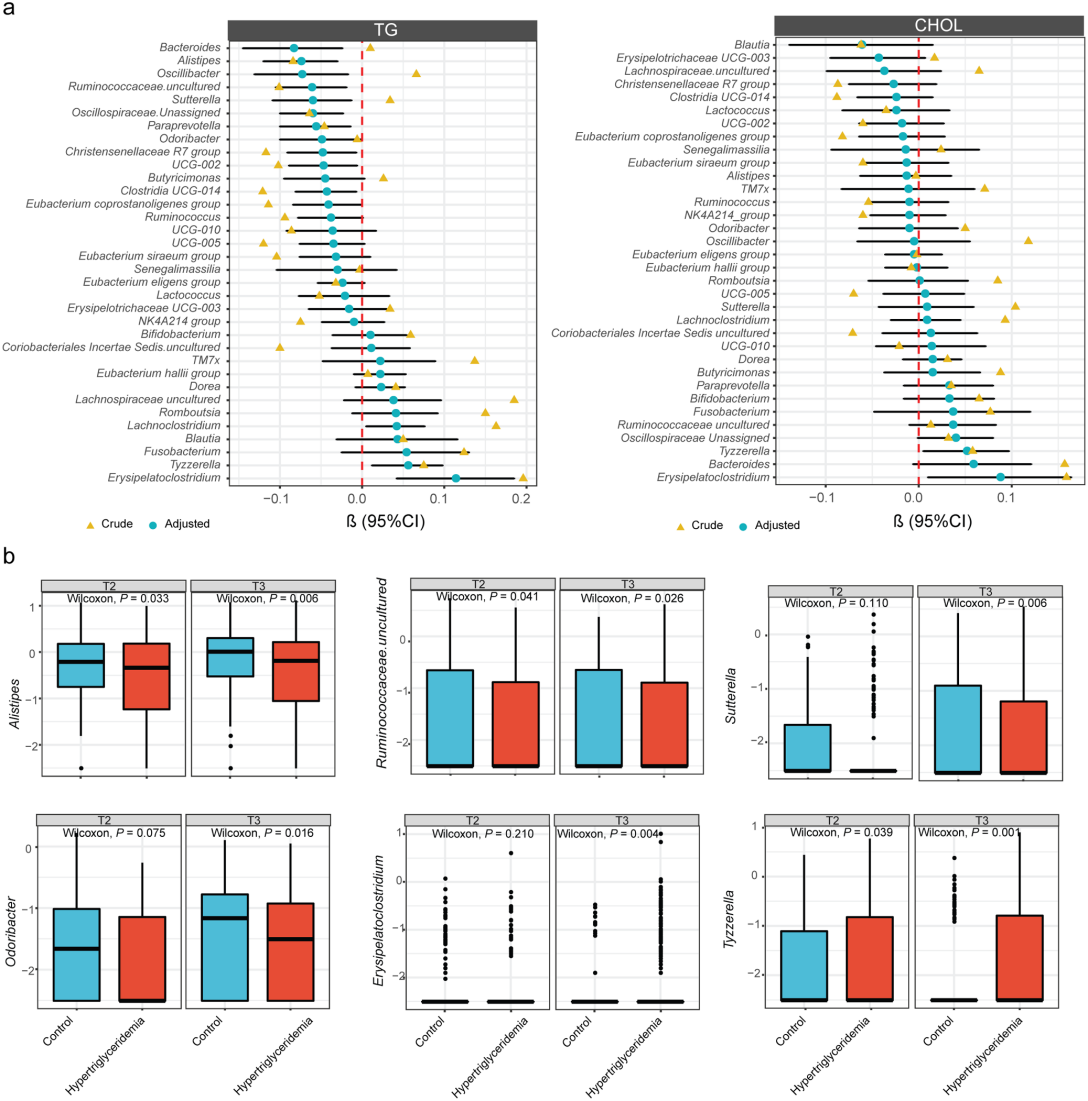
Association between the gut microbiota and dyslipidemia. **a** Genera associated with TG and CHOL levels with and without covariate adjustment. Covariates included in all models were maternal age, prepregnancy BMI, parity, and sampling time. Genera shown had covariate-adjusted FDR < 0.2 in one of the results identified by different periods. Symbols (yellow and blue) showed the beta coefficient, and error bars represented standard error. **b** Differential genera between hypertriglyceridemia and non-hypertriglyceridemia groups. The center line denoted the median. The boxes covered the 25th and 75th percentiles, and the whiskers extended to the most extreme data point, which was no more than 1.5 times the length of the box away from the box. Points outside the whiskers represented outlier samples. *P* values in the plots showed *P* obtained from the Wilcoxon rank-sum test.

To investigate the association of gut microbiota community with different types of dyslipidemia during pregnancy, we compared the diversity indexes between healthy women and women with dyslipidemia. Compared to healthy women, Shannon indexes decreased significantly in hypertriglyceridemia at T2 (Supplementary Fig. 3a). A significant overall difference in gut microbiome community composition in dyslipidemia groups was shown at T2 (PERMANOVA test, *P* = 0.038). Shannon index was negatively associated with TG levels at T2 but not at T3 (Supplementary Fig. 3b). In addition, the microbial structure was different among dyslipidemia groups at T2 and T3 (Supplementary Fig. 3c). We assessed the association of gut microbiota community abundance with dyslipidemia during pregnancy using a linear mixed model (LMM). We identified several genera associated with TG levels during pregnancy at the genus level. The results from LMM and MaAslin indicated that *Bacteroides*, *Paraprevotella*, *Alistipes, Christensenellaceae R7 group*, *Clostridia UCG-014*, and *UCG-002* were negatively associated with TG levels during pregnancy (Fig. 4a). The results were consistent during each pregnancy period (Supplementary Fig. 4a, b). In addition, we compared the abundance of identified key genera among dyslipidemia groups. The identified genera showed the same trend with enrichment in the healthy women (Fig. 4b, Supplementary Fig. 4c).

To investigate specific gut microbiota taxa in mid-pregnancy associated with lipid levels in late pregnancy, we adopted a generalized regression analysis adjusted for confounding factors. Several genera were identified as being associated with TG and CHOL levels (Supplementary Fig. 4b, c). In those genera, consistent with the LMM methods, *Alistipes* and *Christensenellaceae R7 group* were significantly enriched in healthy women (Supplementary Fig. 4c). Importantly, these genera associated with lipid levels were stable during gestation.

**Fig. 5:**
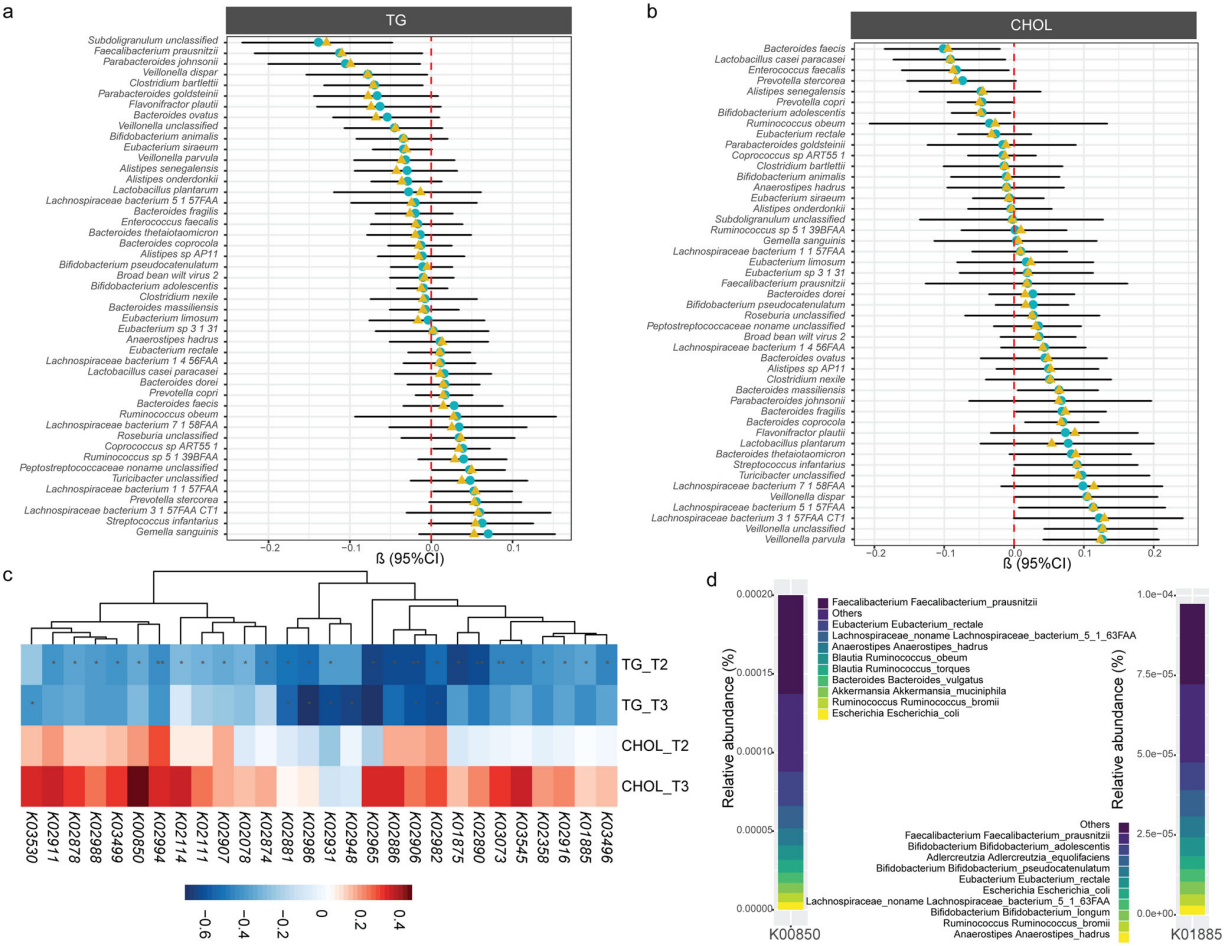
Association of the gut microbiota composition and KOs abundance with lipid levels. **a** Association of key species with TG levels at T2. **b** Association of key species with CHOL levels at T2. **c** Significant KOs associated with lipid levels during pregnancy. **d** Taxa contributing to KOs significantly associated with lipid levels.

To further identify species biomarkers and potential functional pathways affecting lipid levels, we performed a metagenomic analysis at T2. Several species were identified to be significantly associated with TG levels at T2 (Fig. 5a). Among those species identified at T2, *Parabacteroides johnsonii, Coprococcus* sp.ART55-1*, Clostridium bartlettii, Peptostreptococcaceae noname unclassified, Veillonella dispar* also showed consistent results at T3 (Supplementary Fig. 5a). Species associated with TG levels belonged to the genera that were significantly associated with TG levels at T2, such as *Parabacteroides johnsonii, Bacteroides ovatus* and *Lachnospiraceae bacterium 1 1 57FAA*. Similarly, several species were significantly associated with CHOL levels at T2 (Fig. 5b). Among those identified, *Bifidobacterium adolescentis, Bacteroides coprocola, Bacteroides faecis, Bacteroides fragilis, Veillonella parvula, Veillonella unclassified* were consistent at both T2 and T3. In contrast, the various *Bacteroides* species revealed different, even opposite results (Supplementary Fig. 5b).

At the functional level, 18 KEGG orthologous (KOs) were identified to be associated with lipid levels during pregnancy (Fig. 5c), mainly significantly associated with TG levels. Among those KOs associated with TG levels, K00850 and K01885 belonged to 6-phosphofructokinase and glutamyl-tRNA synthetase, respectively. Consistent with the identified species, the taxa contributing to K00850 and K01885 were mainly composed of *Faecalibacterium* species (Fig. 5d). Pathway-enrichment analysis of KOs indicated that immune-associated pathways (IL-17 signaling pathway and Th17 cell differentiation) were depleted in the hyperlipidemia group. At the same time, inositol phosphate metabolism and mineral absorption were enriched (Supplementary Fig. 5c, d).

To further investigate whether the biochemical profile can mediate the microbial effect on dyslipidemia, we performed a mediation analysis and revealed 13 mediation links (mediation analysis, *P* < 0.05, Supplementary Fig. 6a). Interestingly, most of these linkages were related to uric acid. As shown in Supplementary Fig. 6b, *Alistipes* might contribute to the decreased risk of dyslipidemia by affecting serum levels of retinol-binding protein (proportion: 8.8%, mediation analysis, *P* < 0.05) and uric acid (proportion: 3.7%, mediation analysis, *P* < 0.05). Furthermore, *Bacteroides* may contribute to decreased dyslipidemia by affecting uric acid (proportion: 11.3%, mediation analysis, *P* < 0.05).

### Microbial and clinical markers of discriminating dyslipidemia during pregnancy

**Fig. 6:**
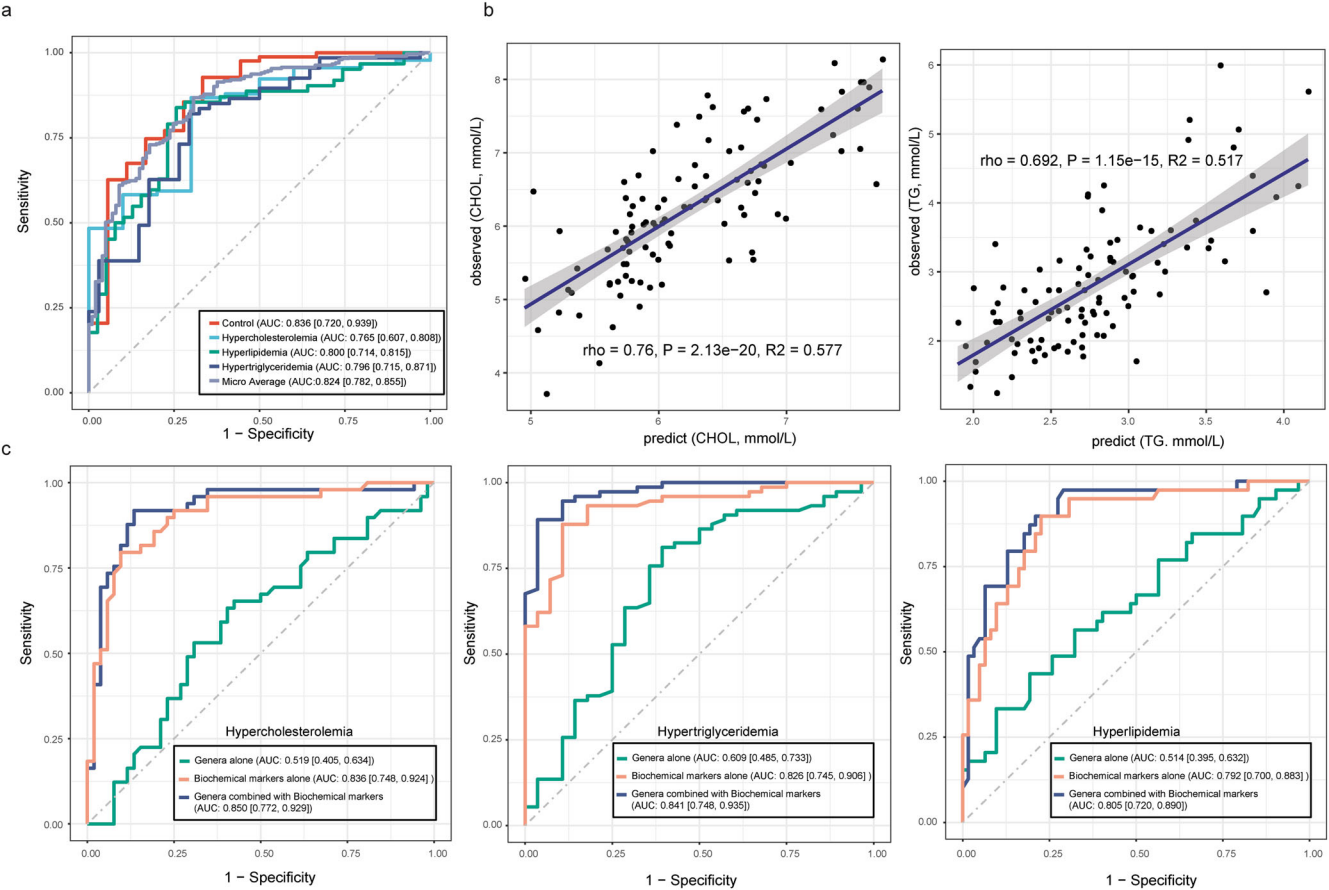
Prediction of dyslipidemia using gut microbiome and biochemical tests and cross-validation. **a** Prediction performance of dyslipidemia using random forest classifiers trained on the genus-level taxonomic profiles and biochemical test levels of the gut microbiome. Numbers in parentheses represented the 95% confidence interval of the AUC. **b** Prediction performances of TG levels and CHOL levels using random forest regression models trained on the genus-level taxonomic profiles of the gut microbiome and biochemical test levels. *P* values in the plots showed *P* obtained from the linear regression. **c** Prediction performances of dyslipidemia groups using random forest classifiers trained on various profiles of the gut microbiome and biochemical test levels. Numbers in parentheses represented the 95% confidence interval of the AUC.

We employed a multi-level random forest classifier to discriminate dyslipidemia patients based on the potential associations of the mid-pregnancy gut microbiota community and the lipid levels in late pregnancy. We trained the model for classifying healthy and dyslipidemia groups using cross-validation with random training in 80% of the samples and 100 bootstrap iterations based on several vital genera. The micro-average area under the curve (AUC) value for selected genera combined with biochemical markers to predict dyslipidemia was 0.824 (95% CI: 0.782–0.855), which improved the prediction performance of biochemical markers alone (Fig. 6a). The AUC values for hypertriglyceridemia, hypercholesterolemia, and hyperlipidemia were 0.796 (95% CI: 0.715–0.817), 0.765 (95% CI: 0.607–0.878) and 0.800 (95% CI: 0.714–0.865), respectively (Fig. 6c). Except for hypercholesterolemia, the F1 scores for other dyslipidemia types were larger than 0.6 (Supplementary Fig. 7a). To further identify the dyslipidemia-type specific biomarkers, we constructed a two-level random forest classifier that produced the AUC values of 0.841 (95% CI: 0.748–0.935) for hypertriglyceridemia, 0.850 (95% CI: 0.772–0.929) for hypercholesterolemia, and 0.805 (95% CI: 0.720–0.890) for hyperlipidemia (Fig. 6c), respectively, which is consistent with the dyslipidemia classifier. The combination of biochemical markers and selected gut microbiome biomarkers increased the range of AUCs from 0.810 to 0.850 (Fig. 6c). Similarly, we constructed a random forest regression model to predict lipid levels in late pregnancy. The R^2^ was 0.516 and 0.568 for TG and CHOL levels, respectively (Fig. 6b). The prediction performances were summarized in Supplementary Table 3. The dominant factors contributing to the prediction model based on gut microbiota and clinical markers were similar. Apart from lipid levels, *Alistipes* and *Christensenellaceae R7 group* were the main influencing genera (Supplementary Fig. 7b).

We also built prediction models based on species level to investigate whether gut microbiota at species level combined with biochemical tests. However, there was no significant improvement in the performance of species combined with biochemical markers compared to biochemical markers alone (Supplementary Fig. 7c, d).

## Discussion

In this study, we evaluated the dynamic changes of gut microbiota during pregnancy and its association with dyslipidemia. The gut microbiota community changed significantly with decreased alpha diversity, increased beta diversity, and dramatic changes in several taxa from T2 to T3. Using dyslipidemia and lipid levels at T2 and T3 as the outcomes, we identified microbiome biomarkers associated with dyslipidemia groups. The association of gut microbiome with dyslipidemia was further confirmed by the random forest prediction model, which suggested that the mid-pregnancy gut microbiome combined with biochemical markers has the potential to predict dyslipidemia in late pregnancy.

In normal pregnancy, the gut bacterial load has been reported to increase^19^. Limited studies investigated microbial diversity and abundance changes during normal pregnancy, and the results were conflicting. We found that gut microbiota changed dramatically from T2 to T3 with an increased Shannon index, a vast expansion of diversity, an overall increase in Bacteridota, and a decrease in Firmicutes, which is in agreement with the previous report^13^. Moreover, evidence indicated that as the fetus developed in the third trimester, the total maternal gut microbiota load increased, and the beta diversity among pregnant individuals increased, whereas the richness and evenness of individuals decreased^20^. However, conflicting results from another study indicated that the microbiota community, taxonomic composition and diversity at all four body sites remained remarkably stable during gestation^21^. An extensive population-level survey of the gut microbiota that included 1479 pregnant women demonstrated a relatively stable microbiota throughout gestation^22^. The inconsistent observations of these studies may be due to the complexity and variability of the gut microbiota and the limited sample sizes, as well as the physical^23^ or psychological^24^ status of pregnant women before and during pregnancy. Further studies with larger sample sizes, standardized study designs, and sample collection criteria are needed to validate the result.

We compared the gut community structure between dyslipidemia types and their healthy counterparts during pregnancy and found that the community structure was significantly different between groups at T2 but not at T3, accompanied by increased lipid levels and prevalence of dyslipidemic women, indicating patients with dyslipidemia have a unique gut microbiota structure during pregnancy. In addition, we found that pregnant women with dyslipidemia had lower alpha diversity compared to women with normal lipid levels. Several genera changed dramatically during gestation. However, genera we identified significantly associated with lipid levels, including *Alistipes, Bacteroides, Paraprevotella, Christensenellaceae R7 group, Clostridia UCG-014*, and *UCG-002* were stable during pregnancy. Those identified genera were found to be associated with blood biochemical profiles, and most of these linkages were related to uric acid. Previuos data indicated that gut microbiota participated in uric acid metabolism^25^, which modulated lipid metabolism^26^. *Alistipes* is a propionate producer that expresses methylmalonyl-CoA epimerase^27^ and an acetate producer^28^. The unique process of fermenting amino acids allows *Alistipes* to play an essential role in inflammation and disease^29^. Since previous studies have shown that short-chain fatty acids (SCFAs) have anti-inflammatory mechanisms, it may be that the decrease of *Alistipes* contributes to the reduction of SCFA, where SCFAs are not only involved in lipid metabolism as substrates but also act as regulators to modulate lipid metabolism^30^. The *Christensenellaceae R7 group* has also been consistently reported to be negatively related to visceral fat mass and indicated as a marker of lean phenotype^31,32^. Moreover, *Bacteroides*, *Alistipes*, and *Paraprevotella* members, belonging to the Bacteroidetes phylum, could play a role in adiposity modulation through the production of two SCFAs, acetate and propionate^33^. These identified genera shared similar functional pathways, including inflammatory mechanisms, which indicated the potential mechanism of gut microbiota affecting lipid profile.

We further investigate the role of gut microbiota at the species level in the development of dyslipidemia during pregnancy. Even when belonging to the same genus, different species can exert diverse effects on the lipid profile. *Bacteroides faecis* was found to be associated with reduced CHOL. Other species, such as *Bacteroides coprocola* and *Bacteroides fragilis*, were positively associated with lipid levels, and both of them have been reported to promote intestinal inflammation^34^. Functional analysis of the gut microbiome across pregnant women at T2 revealed the significant enrichment of KOs involved in carbohydrate metabolism and immune-associated pathways. Previous findings have indicated that inflammation contributes to dyslipidemia in both the fasting and postprandial states^35^, reflecting the possibility that the gut microbiota may influence lipid profiles during pregnancy through inflammation.

We found that the gut microbiota during mid-gestation can predict the risk of dyslipidemia to some extent and performed well when combined with clinical variables. Predictive models using genus-level microbiota data performed better than species, and no single species could be considered a consistent biomarker. This can be explained by the low relative abundance of key species levels, resulting in a less distinct differentiation of dyslipidemia groups and reduced predictive power. In addition, these critical species biomarkers we identified have similar functions as a whole genus group.

In addition to the large sample size of the cohort, our study has multiple advantages. Firstly, we combined amplicon and metagenomic sequencing analysis of longitudinal microbiome data for the participants to assess the association of gut microbiota and dyslipidemia during pregnancy, which ensured that we explored potential functional pathways by which the gut microbiota affects lipid profile. In addition, based on the selected taxa, we developed a microbiota-based prediction model, including biochemical biomarkers, which ensured that we evaluated the risk of dyslipidemia in the early stage of pregnancy. Although we proposed the potential application of gut microbiome-based therapeutics for patients with dyslipidemia, several limitations remained in our study. Firstly, our cohort is single-centered, and the results should be validated in multiple cohorts in different populations. Moreover, future studies should consider the complex interactions between clinical factors and the gut microbiome in the therapeutic process. In addition, current methods for analyzing the gut microbiome allow for in-depth investigation of taxonomic and functional aspects of the gut microbial community but lack the ability to characterize the microbiome immunologically, which should be further studied in future studies. This study adds to our understanding of the role of the gut microbiome in dyslipidemia in pregnancy and illuminates the complexity of the microbiome’s role in human disease.

## Methods

### Study design

Participants were excluded according to the following criteria: (1) previously diagnosed with diabetes mellitus or GDM, thyroids diseases, intrahepatic cholestasis of pregnancy, tumor, eclampsia, or HBV; (2) antibiotics usage within three months prior to fecal sample collection; (3) multiple pregnancies, and (4) pregnancy with artificial reproductive technology (ART). We excluded women without lipid levels and fecal samples missing during pregnancy at T2 or T3 time points. Finally, 513 pregnant women remained for further analysis. The flow diagram for selecting study subjects is shown in Fig. 1b. Firstly, 16S rRNA sequencing of stool samples from two-time points was performed to explore the dynamics of the gut microbiota and to investigate the potential predictive potential of the gut microbiota at the genus level. To further identify and reveal the functional pathways by which the gut microbiota affects lipid profiles and to determine whether critical species associated with lipid profiles can predict the risk of dyslipidemia, metagenomic sequencing was performed at T2.

Demographic information on the participants was collected through a structured questionnaire, and clinical records, including biochemical data of participants, were extracted from the hospital information systems. The length of gestation was assigned based on the self-reported date of the last menstrual period (LMP).

Dyslipidemia was diagnosed according to the guidelines of the American Heart Association (AHA)^36^: CHOL levels ≥6.22 mmol/L (240 mg/dL), TG levels ≥ 2.26 mmol/L (200 mg/dL), LDL-C levels ≥ 4.1 mmol/L (160 mg/dL), or HDL-C levels < 1.0 mmol/L (40 mg/dL). Based on the Chinese adult dyslipidemia prevention and treatment guidelines (revised in 2016)^37^, we divided dyslipidemia into hypercholesterolemia, hypertriglyceridemia, and hyperlipidemia according to the levels of TG and CHOL levels in the participants. We considered it hyperlipidemia only when both CHOL and TG levels were above the threshold.

All study procedures were reviewed and approved by the institutional review board at Nanjing Maternity and Child Health Care Hospital. Written informed consent was provided by all participants.

### DNA extraction for 16S rRNA gene sequencing and taxonomy profiling

Bacterial total DNA was extracted from 180 to 220 mg of feces using the QIAamp Fast DNA Stool Mini Kit (QIAGEN, Germany) in a sterile environment. Variable regions V3-V4 of the 16 S rRNA gene were amplified from fecal DNA extracts using the primers 338F (5’-ACTCCTACGGGAGGCAGCAG-3’) and 806R (5’-GGACTACHVGGGTWTCTAAT-3’). Purified amplicons were pooled in equimolar and paired-end sequenced on an Illumina MiSeq PE250 platform platform (Illumina, San Diego, USA) according to the standard protocols by Majorbio Bio-Pharm Technology Co. Ltd. (Shanghai, China). Reactions were performed under the following conditions: 94 °C for 2 min, 30 cycles, 94 °C for 20 s, 60 °C for 20 s, 72 °C for 30 s, and a final step of 72 °C for 10 min. DNA concentration was measured with Nanodrop 2000, and purity was quantified. 1% agarose gel electrophoresis was used to determine the integrity of the DNA. The amplification results were sequenced on the Illumina Miseq PE250 platform. Sequence quality control was performed with QIIME2. The paired-end Fastq files were truncated, filtered, denoised, and merged using the dada2 pipeline^38^. Single nucleotide exact amplicon sequence variants (ASVs) were obtained with 100% sequence homology, and taxonomy was assigned with reference to Silva v128^39^ (www.arb-silva.de; release 138). Contaminated sequences (*n* = 131 from 39, 691 total ASVs) were identified and removed using the *decontam* package (frequency methods, default parameter (0.1))^40^. Alpha diversity and β-diversity were calculated with the *vegan* package, with all the samples rarefied to the lowest sequence depth 32,007 (range from 32,007 to 120,183). Genera with relative abundance ≥0.01% across all samples collected and existing in ≥10% of samples (98 from 457 genera) were left for the downstream analysis.

### DNA extraction for metagenomic shotgun sequencing

Total genomic DNA was extracted from feces samples using the E.Z.N.A.® Soil DNA Kit (Omega Bio-tek, Norcross, GA, U.S.) according to the manufacturer’s instructions. The concentration and purity of the extracted DNA were determined with TBS-380 and NanoDrop2000, respectively. DNA extract quality was checked on 1% agarose gel. DNA extract was fragmented to an average size of about 400 bp using Covaris M220 (Gene Company Limited, China) for paired-end library construction. The paired-end library was constructed using NEXTflexTM Rapid DNA-Seq (Bioo Scientific, Austin, TX, USA). Adapters containing the full complement of sequencing primer hybridization sites were ligated to the blunt-end of fragments. Paired-end sequencing was performed on Illumina NovaSeq (Illumina Inc., San Diego, CA, USA) at Majorbio Bio-Pharm Technology Co., Ltd. (Shanghai, China) using NovaSeq Reagent Kits according to the manufacturer’s instructions (www.illumina.com).

### Shotgun metagenomic sequencing and taxonomic and functional profiling

We randomly selected 30% of the samples (*n* = 154) for further metagenomic sequencing. After filtering samples with low quality (*n* = 13), 141 samples finally remained for metagenomic sequencing. Paired-end sequencing was performed on Illumina NovaSeq (Illumina Inc., San Diego, CA, USA) at Majorbio Bio-Pharm Technology Co., Ltd. (Shanghai, China) using NovaSeq Reagent Kits according to the manufacturer’s instructions (www.illumina.com). From the raw metagenomic reads, quality control was performed using the KneadData (v0.7.4) toolkit with default parameters to remove Illumina adapters and reads that were less than 50 bp in length, had an average mass value of less than 20. Trimmed reads aligned to the human genome (GRCh37/hg19) were further removed using the KneadData integrated Bowtie2^41^ tool (version 2.4.2) to avoid human gene contamination. After that, quality control was conducted using the FastQC toolkit (version 0.11.9). The MetaPhlAn2^42^ tool (version 2.7.2) was used to analyze the quantitative profiling of the taxonomic composition of the metagenome, whereas HUMAnN2^43^ (version 0.11.1) was used to profile genes encoding microbial biochemical pathways, which integrated with the DIAMOND alignment tool (version 0.8.22), the uniref90 protein database (version 0.1.1), and the ChocoPhlAn pangenome database (version 0.1.1). After this step, we identified 549 species, 503 KEGG pathways, and 8416 distinct KOs in 141 samples from the participants at T2. Specifically, species were filtered to remove markers with low overall abundance (<0.01%) and existing in <10% of samples. Functional profiles (KOs) were preprocessed to remove abundance <1 × 10^−6^. KEGG pathways were filtered to remove those with abundance <0.01% and existing in <10% of samples. After the filtration, 176 species, 150 KOs, and 268 KEGG pathways were left for the downstream analysis.

### Microbiome co-abundance groups network construction

Genera with relative abundance ≥0.01% across all samples collected and existing in ≥10% of samples (98 from 457 genera) were used to construct co-abundance groups (CAGs) using the Ward clustering algorithm based on Kendall’s correlation coefficients with the *made4* package. Networks were constructed at T2 and T3 separately. The correlation of the genera in constructed CAGs was visualized by Cytoscape 3.7.1 software. Driver genera from T2 to T3 were constructed based on microbial correlation coefficients using the NetShift tool^44^ (https://web.rniapps.net/netshift) based on the networks constructed at both time points. Neighbor shift (NESH) scores and betweenness centrality (BC) measures were used to identify the driver taxa responsible for the correlation change in the two networks. The threshold set for the correlation coefficient was 0.3.

### Qualification and Statistical analysis

The analysis was conducted in four parts: (1) to characterize the dynamic change of the microbiota and lipid levels during pregnancy; (2) to determine the key gut microbiota taxa affecting the lipid levels during pregnancy; (3) to explore the potential functional pathway the key taxa affecting lipid levels and (4) to predict dyslipidemia during pregnancy using key microbiota taxa combined with correlated biochemical data information.

We used a paired Wilcoxon rank sum test to assess the change in maternal lipid levels between the T2 and T3 trimesters for non-normally distributed data. Similarly, the dynamic change of alpha diversity indexes from T2 to T3 was compared by the Wilcoxon rank sum test. Furthermore, the dynamic β-diversity changes of maternal microbiota from T2 to T3 were assessed by permutational analysis of variance (PERMANOVA) based on an unweighted UniFrac distances matrix, as implemented in the ADONIS test. Dirichlet multinomial mixtures (DMM) clustering was used to explore the community typing of microbial community profiling at the genus level during pregnancy. DMM bins samples on the basis of microbial relative abundance levels. We determine the optimal number of clusters based on the lowest Laplace approximation score^45^. We compared the difference in lipid levels among clusters using the Kruskal-Wallis test and aimed to identify the dominant genera affecting lipid levels.

Associations of gut microbiota community structure and compositional abundance over time with lipid levels were tested in the longitudinal cohort using a linear mixed effect model with time as the fixed effect and subject ID as a random effect using the lme-function in the *nlme* package^46^. The Benjamini–Hochberg procedure was used to correct for multiple comparisons^47^, and a corrected *P* < 0.2 was considered significant. MaAsLin analysis was also performed to determine the association using *Maaslin2* package. Subject ID was used as a random effect. The following variables were used as fixed effects in addition to the variables of interest for every analysis: pre-pregnancy BMI, parity, age, and sampling time. Only genera both significant in these two analyses were considered as significantly associated with lipid levels during pregnancy. To explore whether species are associated with lipid levels and which functional pathways played an essential role, multiple linear regression and Lefse analyses were conducted. To investigate the mediation linkages between microbial features, biochemical profile, and dyslipidemia, we performed mediation analysis using the R package *mediation*.

In all analyses, abundance information for genera and species were log10-transformed after adding a pseudocount of 1 × 10^−5^ to avoid nonfinite values. KOs were log10-transformed after adding 1 × 10^−7^ as a pseudocount.

To determine how well gut microbiota and biochemical test data predicted dyslipidemia, multi-class random forest classification was performed using the *randomForest* package. Specifically, we develop two-class random-forest classifier models to evaluate the prediction performance of gut microbiota combined with biochemical test levels predicting dyslipidemia groups (Hypercholesterolemia, hypertriglyceridemia, and hyperlipidemia). In brief, the model was trained on key genera associated with lipid levels, associated biochemical tests, and key genera combined with associated biochemical biomarkers. The model was built using the following parameters: growing 500 trees and n/3 features randomly sampled at each split, where n represents the number of features. The model was further validated with 100 bootstrap cross-validations by splitting datasets by the ratio of 4:1 (training set: testing set). The accuracy of the classifiers was determined by calculating the area under the receiver operating characteristic curve (AUC). Sensitivity, specificity, and F1 scores were used to show the performance of the prediction model. The significance of differences in the accuracy of the derived classifiers and confidence intervals were assessed using the bootstrap method of roc.test in the *pROC* package. The Gini index was used to measure the features’ importance. In addition, we built random forest regression models to predict the lipid levels at T3. R^2^ was used to show the performance of the regression model. The mean squared error (MSE) was used to measure the quality of predictors in random forest regression models.

All statistical analyses were conducted using RStudio software (version 1.2.5019). A two-tailed *P* < 0.05 was considered statistically significant.

### Reporting summary

Further information on research design is available in the Nature Research Reporting Summary linked to this article.

## Supplementary information

Supplementary Materials

Reporting Summary

## Data Availability

The raw sequence data reported in this paper have been deposited in the Genome Sequence Archive in National Genomics Data Center, China National Center for Bioinformation / Beijing Institute of Genomics, Chinese Academy of Sciences (GSA: CRA010129) that are publicly accessible at https://ngdc.cncb.ac.cn/gsa.

## Code availability

The code (R scripts) is available at: https://github.com/Xia-lab-NMU/GMD_study.
