# Supplementary material for: Ecological change of the gut microbiota during pregnancy and progression to dyslipidemia
Source: NPJ Biofilms Microbiomes. 2023 Apr 3;9:14. doi: 10.1038/s41522-023-00383-7 (PMC10070613; doi:10.1038/s41522-023-00383-7)
Supplement: Supplementary file 1 — Supplementary Materials [file 41522_2023_383_MOESM1_ESM.pdf]

Supplementary Materials

Ecological change of the gut microbiota during pregnancy and progression to dyslipidemia

Xu Yang[1,2†], Mingzhi Zhang[1,2†], Yuqing Zhang[3], Hongcheng Wei[1,2], Quanquan Guan[1,2],Chao Dong[1,2], Siting Deng[1,2], Hein Min Tun[4,5,6], Yankai Xia[1,2*]

[1]State Key Laboratory of Reproductive Medicine, School of Public Health, Nanjing Medical University, Nanjing, China

[2]Key Laboratory of Modern Toxicology of Ministry of Education, School of Public Health, Nanjing Medical University, Nanjing, China

[3]Women's Hospital of Nanjing Medical University, Nanjing Maternity and Child Health Care Hospital, Nanjing, China

[4]The Jockey Club School of Public Health and Primary Care, Faculty of Medicine, The Chinese University of Hong Kong, Hong Kong SAR, China

[5]Li Ka Shing Institute of Health Sciences, The Chinese University of Hong Kong, Hong Kong SAR, China

[6]HKU-Pasteur Research Pole, School of Public Health, Li Ka Shing Faculty of Medicine, The University of Hong Kong, Hong Kong SAR, China

†These authors contributed equally to this work.

*Corresponding author: Yankai Xia, yankaixia@njmu.edu.cn

Dr. Yankai Xia

State Key Laboratory of Reproductive Medicine, School of Public Health, Nanjing Medical University, No.101 Longmian Road, Nanjing 211166, China. E-mail: yankaixia@njmu.edu.cn; Phone: +86-25-86868425

Index Page

Supplementary Table 1 2
Supplementary Table 2 4
Supplementary Table 3 6
Supplementary Figure 1 7
Supplementary Figure 2 8
Supplementary Figure 3 9
Supplementary Figure 4 10
Supplementary Figure 5 11
Supplementary Figure 6 12
Supplementary Figure 7 13

Supplementary Table 1. Baseline characteristics of the study population in the second trimester (T2)

Characteristic	Overall (n=513)	T2				P value
		Control (n=241)	Hypercholesterolemia (n=88)	Hyperlipidemia (n=66)	Hypertriglyceridemia (n=126)	
Age [years, mean (sd)]	29.2 (3.38)	28.6 (3.04)	29.1 (3.12)	29.8 (3.56)	29.8 (3.88)	<0.001
prepregnancy BMI [kg/m2, mean (sd)]	21.0 (2.41)	20.7 (2.22)	20.4 (2.47)	21.6 (2.72)	21.5 (2.40)	<0.001
BMI<18.5	65 (12.7%)	34 (14.1%)	15 (18.8%)	7 (10.6%)	9 (7.14%)	
18.5≤BMI＜25	415 (80.9%)	197 (81.7%)	60 (75.0%)	52 (78.8%)	106 (84.1%)	
BMI≥25	33 (6.43%)	10 (4.15%)	5 (6.25%)	7 (10.6%)	11 (8.73%)	
Parity [No. (%)]						0.001
0	380 (74.1%)	194 (80.5%)	60 (75.0%)	42 (63.6%)	84 (66.7%)	
≥1	133 (25.9%)	47 (19.5%)	20 (25.0%)	24 (36.4%)	42 (33.3%)	
Education [No. (%)]						0.209
<High School	42 (8.19%)	28 (11.6%)	6 (7.50%)	3 (4.55%)	5 (3.97%)	
High school-Bachelor degree	395 (77.0%)	178 (73.9%)	59 (73.8%)	53 (80.3%)	105 (83.3%)	
>Bachelor degree	76 (14.8%)	35 (14.5%)	15 (18.8%)	10 (15.2%)	16 (12.7%)	
Income [No. (%)]						0.923
<200,000	162 (56.6%)	78 (56.9%)	25 (52.1%)	24 (68.6%)	35 (53.0%)	
≥200,000	124 (43.4%)	59 (43.1%)	23 (47.9%)	11 (31.4%)	31 (47.0%)	
Drinking history [No. (%)]						0.594
No	488 (95.1%)	231 (95.9%)	75 (93.8%)	63 (95.5%)	119 (94.4%)	
Yes	25 (4.87%)	10 (4.15%)	5 (6.25%)	3 (4.55%)	7 (5.56%)	
Smoking history [No. (%)]						0.25
No	367 (98.4%)	182 (97.3%)	57 (100%)	41 (100%)	87 (98.9%)	
Yes	6 (1.61%)	5 (2.67%)	0 (0.00%)	0 (0.00%)	1 (1.14%)	
Passive smoking history [No. (%)]						0.346
No	128 (35.8%)	69 (38.1%)	18 (34.0%)	15 (34.9%)	26 (32.1%)	
Yes	230 (64.2%)	112 (61.9%)	35 (66.0%)	28 (65.1%)	55 (67.9%)	
CHOL at T2 [mmol/L, mean (sd)]	5.81 (0.91)	5.33 (0.57)	6.90 (0.67)	6.94 (0.54)	5.44 (0.54)	<0.001
CHOL at T3 [mmol/L, mean (sd)]	6.26 (0.98)	5.90 (0.78)	7.27 (0.90)	7.04 (0.85)	5.88 (0.69)	0.127
TG at T2 [mmol/L, mean (sd)]	2.16 (0.71)	1.71 (0.31)	1.79 (0.31)	2.87 (0.53)	2.89 (0.63)	<0.001

TG at T3 [mmol/L, mean (sd)]	2.82 (0.87)	2.43 (0.61)	2.48 (0.62)	3.41 (0.74)	3.49 (0.94)	<0.001
Gender [No. (%)]						0.699
Boys	253 (51.5%)	121 (52.6%)	32 (42.1%)	35 (53.8%)	65 (54.2%)	
Girls	238 (48.5%)	109 (47.4%)	44 (57.9%)	30 (46.2%)	55 (45.8%)	

Supplementary Table 2. Baseline characteristics of the study population in the third trimester (T3)

Characteristic	Overall (n=513)	T3 Control (n=93)	Hypercholesterolemia (n=50)	Hyperlipidemia (n=199)	Hypertriglyceridemia (n=171)	P value
Age [years, mean (sd)]	29.2 (3.38)	29.0 (3.20)	29.5 (3.48)	29.1 (3.43)	29.2 (3.40)	0.853
prepregnancy BMI [kg/m2, mean (sd)]	21.0 (2.41)	20.7 (2.14)	20.7 (1.97)	20.9 (2.53)	21.4 (2.49)	0.015
BMI<18.5	65 (12.7%)	14 (15.1%)	6 (12.0%)	34 (17.1%)	11 (6.43%)	
18.5≤BMI＜25	415 (80.9%)	75 (80.6%)	42 (84.0%)	152 (76.4%)	146 (85.4%)	
BMI≥25	33 (6.43%)	4 (4.30%)	2 (4.00%)	13 (6.53%)	14 (8.19%)	
Parity [No. (%)]						0.049
0	380 (74.1%)	76 (81.7%)	38 (76.0%)	145 (72.9%)	121 (70.8%)	
≥1	133 (25.9%)	17 (18.3%)	12 (24.0%)	54 (27.1%)	50 (29.2%)	
Education [No. (%)]						0.621
<High School	42 (8.19%)	9 (9.68%)	5 (10.0%)	14 (7.04%)	14 (8.19%)	
High school-Bachelor degree	395 (77.0%)	70 (75.3%)	33 (66.0%)	156 (78.4%)	136 (79.5%)	
>Bachelor degree	76 (14.8%)	14 (15.1%)	12 (24.0%)	29 (14.6%)	21 (12.3%)	
Income [No. (%)]						0.385
<200,000	162 (56.6%)	21 (47.7%)	14 (51.9%)	72 (62.6%)	55 (55.0%)	
≥200,000	124 (43.4%)	23 (52.3%)	13 (48.1%)	43 (37.4%)	45 (45.0%)	
Drinking history [No. (%)]						0.321
No	488 (95.1%)	90 (96.8%)	48 (96.0%)	189 (95.0%)	161 (94.2%)	
Yes	25 (4.87%)	3 (3.23%)	2 (4.00%)	10 (5.03%)	10 (5.85%)	
Smoking history [No. (%)]						0.267
No	367 (98.4%)	69 (98.6%)	37 (100%)	144 (99.3%)	117 (96.7%)	
Yes	6 (1.61%)	1 (1.43%)	0 (0.00%)	1 (0.69%)	4 (3.31%)	
Passive smoking history [No. (%)]						0.015
No	128 (35.8%)	31 (47.0%)	14 (41.2%)	48 (34.0%)	35 (29.9%)	
Yes	230 (64.2%)	35 (53.0%)	20 (58.8%)	93 (66.0%)	82 (70.1%)	
CHOL at T2 [mmol/L, mean (sd)]	5.81 (0.91)	5.16 (0.65)	6.35 (0.76)	6.36 (0.76)	5.37 (0.73)	0.401

CHOL at T3 [mmol/L, mean (sd)]	6.26 (0.98)	5.47 (0.56)	6.92 (0.71)	7.04 (0.66)	5.58 (0.62)	0.889
TG at T2 [mmol/L, mean (sd)]	2.16 (0.71)	1.60 (0.41)	1.66 (0.34)	2.33 (0.65)	2.42 (0.75)	<0.001
TG at T3 [mmol/L, mean (sd)]	2.82 (0.87)	1.86 (0.25)	1.95 (0.25)	3.16 (0.68)	3.21 (0.83)	<0.001
Gender [No. (%)]						0.507
Boys	253 (51.5%)	45 (50.6%)	25 (56.8%)	89 (46.4%)	94 (56.6%)	
Girls	238 (48.5%)	44 (49.4%)	19 (43.2%)	103 (53.6%)	72 (43.4%)	

Supplementary Table 3. AUC values of prediction of dyslipidemia using randomforest model using different predictors

Predictor variables	Group	AUC (Median)	LCI	HCI
Genera alone	Control	0.536	0.409	0.659
	Hypercholesterolemia	0.520	0.343	0.691
	Hyperlipidemia	0.515	0.422	0.614
	Hypertriglyceridemia	0.484	0.386	0.590
	micro Average	0.669	0.635	0.700
Biochemical data alone	Control	0.838	0.732	0.935
	Hypercholesterolemia	0.785	0.599	0.899
	Hyperlipidemia	0.799	0.737	0.853
	Hypertriglyceridemia	0.802	0.717	0.875
	micro Average	0.839	0.794	0.879
Genera combiend with biochemical data	Control	0.836	0.720	0.939
	Hypercholesterolemia	0.765	0.607	0.878
	Hyperlipidemia	0.800	0.714	0.865
	Hypertriglyceridemia	0.796	0.715	0.871
	micro Average	0.824	0.782	0.855

Abbrevations: LCI: Lower confidence interval; HCI: Higher confidence interval

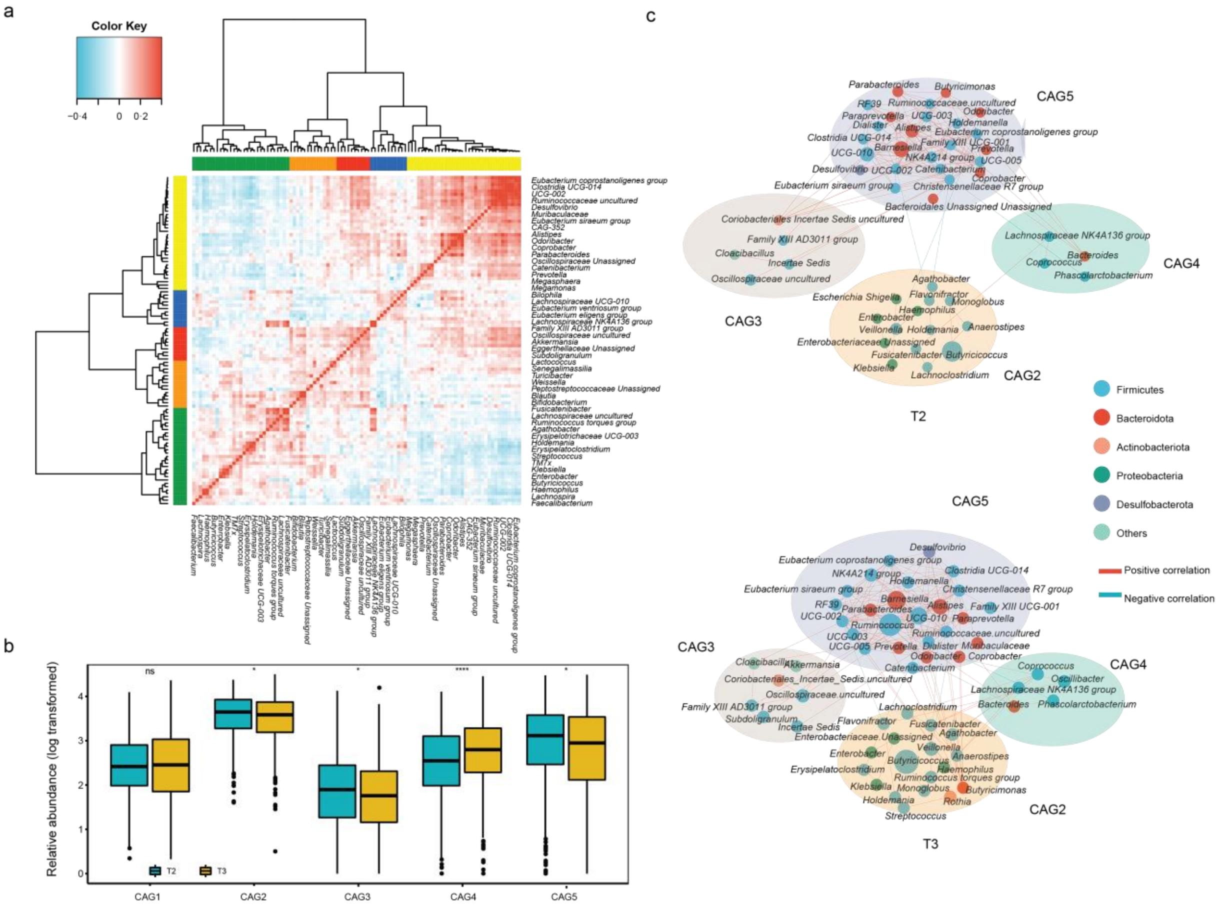

Supplementary Figure 1. Co-abundance groups (CAG) constructed using genera and "drivers" taxa responsible. a: Heatmap of correlation of genera in different clusters assigned with different colors; b: Comparison of relative abundance of CAG abundance during T2 and T3 trimesters. The center line denoted the median. The boxes covered the 25th and 75th percentiles, and the whiskers extended to the most extreme data point, which was no more than 1.5 times the length of the box away from the box. Points outside the whiskers represented outlier samples. Stars with significance in the plots showed P obtained from the Wilcoxon rank-sum test (ns: $P > 0.05$, *: $P < 0.05$, **: $P < 0.01$, ***: $P < 0.001$, ****: $P < 0.0001$); c: Co-abundance of networks of gut microbiota at T2 and T3, respectively. The blue edge meant negatively correlated, and the red edge meant positively correlated. Different colored dots meant different categories.

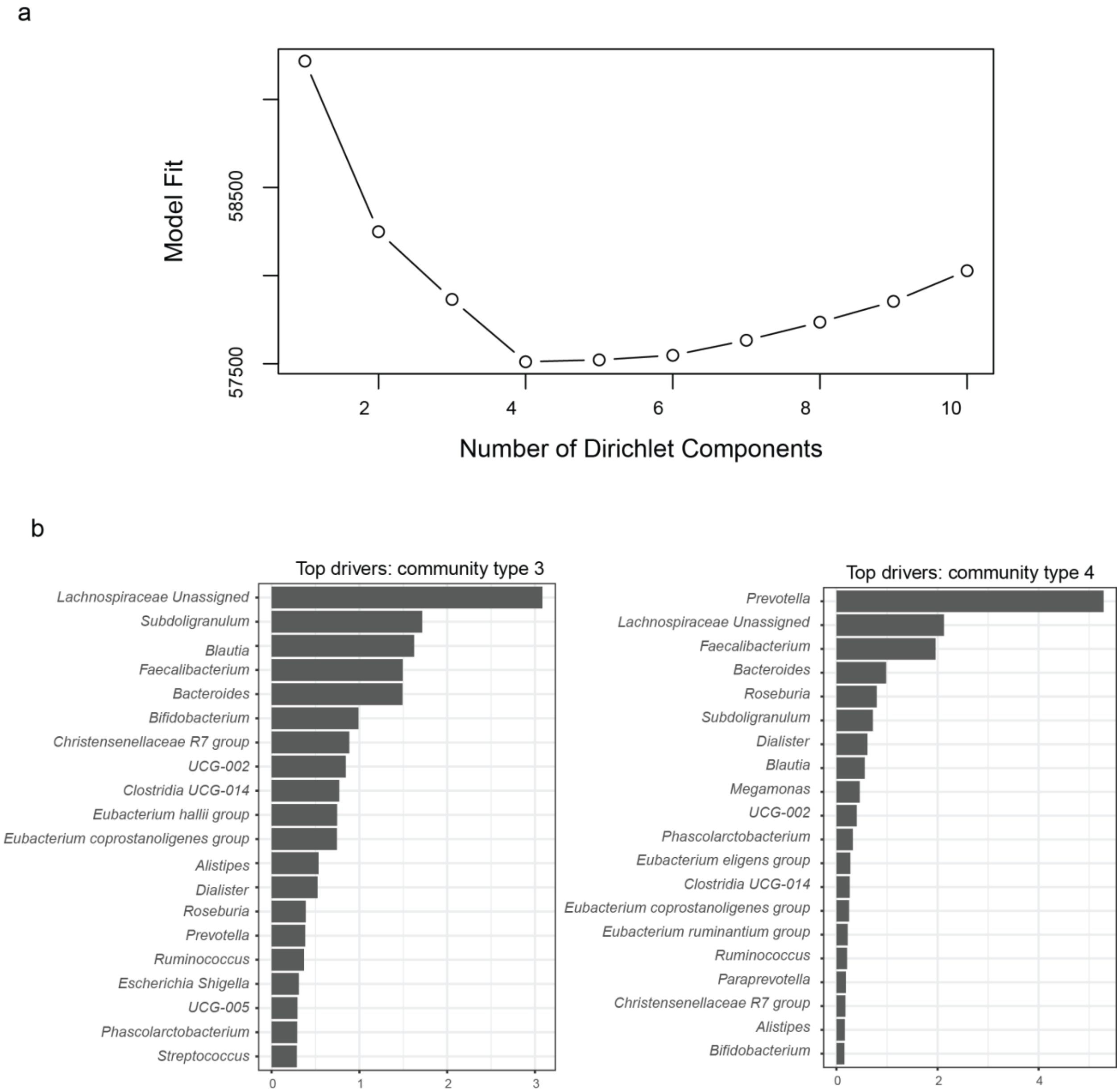

Supplementary Figure 2. The top drivers in four distinct clusters based on lowest Laplace approximation. a. Model performance of the number of dichichlet components; b. The top drivers in distinct clusters 3 and 4.

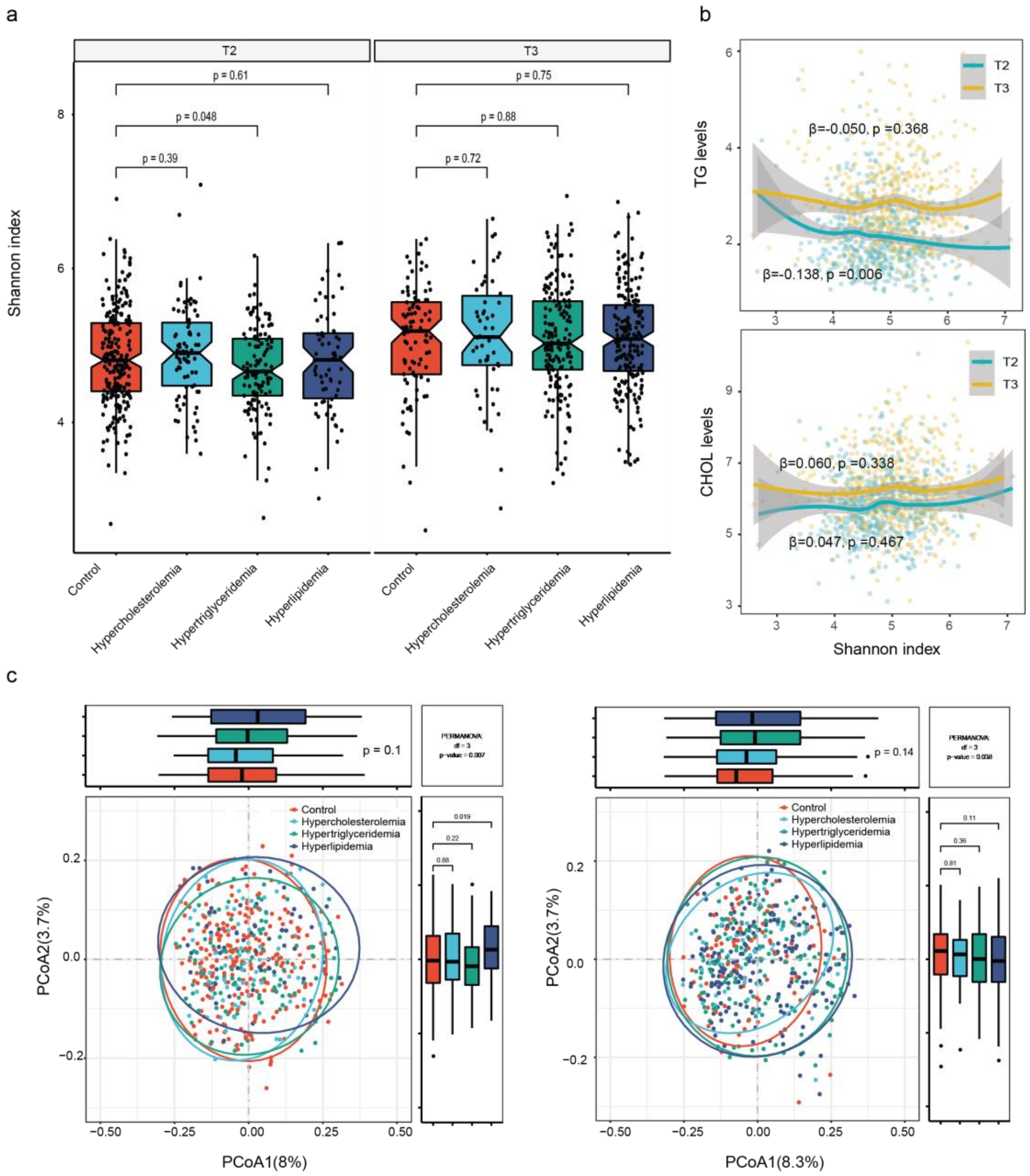

Supplementary Figure 3. Gut microbiota community structure among dyslipidemia groups during pregnancy. a: Boxplot of alpha diversity among dyslipidemia groups. The center line denoted the median. The boxes covered the 25th and 75th percentiles, and the whiskers extended to the most extreme data point, which was no more than 1.5 times the length of the box away from the box. Points outside the whiskers represented outlier samples. *P* values in the boxplots and blank squares showed *P* obtained from the Wilcoxon rank-sum test; b. Association of alpha diversity and lipid levels; c. Unweighted UniFrac principal component analysis of the microbiota of healthy controls and individuals with dyslipidemia. For boxplots, the center line denoted the median. The boxes covered the 25th and 75th percentiles, and the whiskers extended to the most extreme data point, which was no more than 1.5 times the length of the box away from the box. Points outside the whiskers represented outlier samples. *P* values in the boxplots and blank squares showed *P* obtained from the Kruskal-Wallis test and PERMANOVA test, separately.

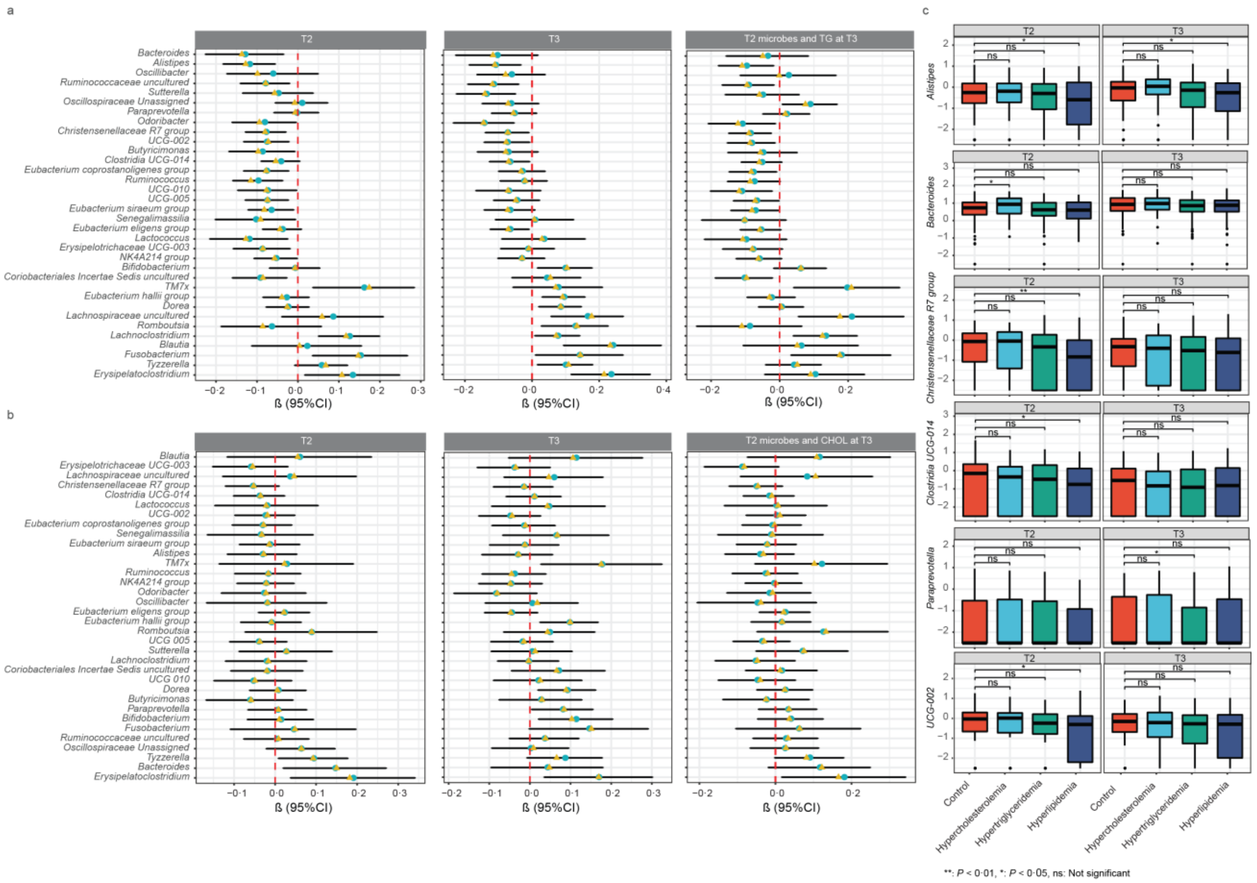

Supplementary Figure 4. Association between the gut microbiota and lipid levels. a. Key genera associated with TG levels at T2 and T3; b. Key genera associated with CHOL levels during pregnancy; c. Comparison of key genera among dyslipidemia groups. The center line denoted the median. The boxes covered the 25th and 75th percentiles, and the whiskers extended to the most extreme data point, which was no more than 1.5 times the length of the box away from the box. Points outside the whiskers represented outlier samples. Stars with significance in the plots showed *P* obtained from the Wilcoxon rank-sum test (ns: *P* >0.05, *: *P* < 0.05, **: *P* < 0.01, ***: *P* <0.001)

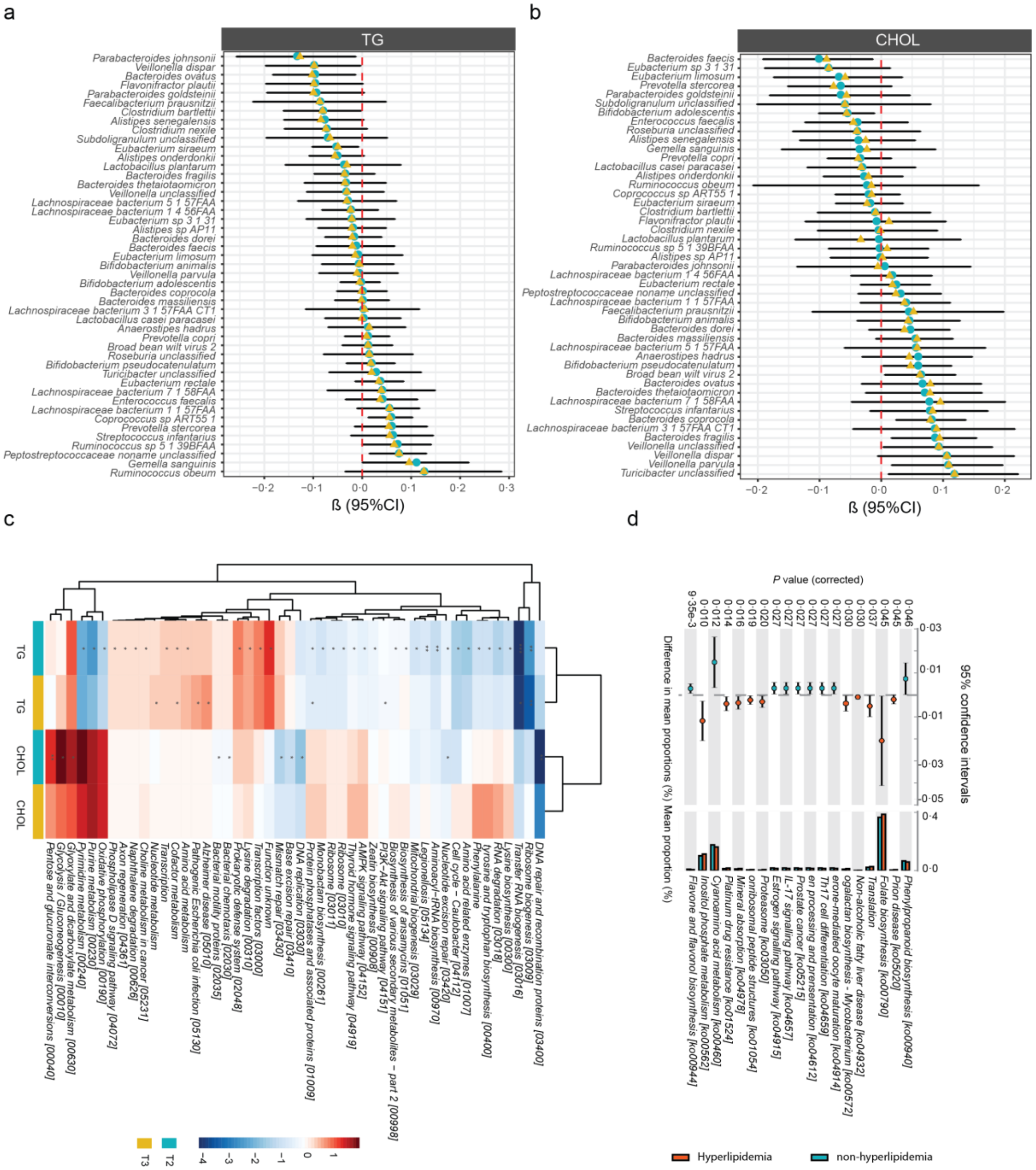

Supplementary Figure 5. Association of the compositional and functional abundance at species level and lipid levels. a. Key species associated with TG levels at T3; b. Key genera associated with CHOL levels at T3; c. Heatmap of key KEGG pathway associated with lipid levels during pregnancy; c. Differential KEGG pathways between hyperlipidemia and non-hyperlipidemia groups during pregnancy. *P* values in the boxplots and blank squares showed *P* obtained from the Wilcoxon rank-sum test with fdr-corrected.

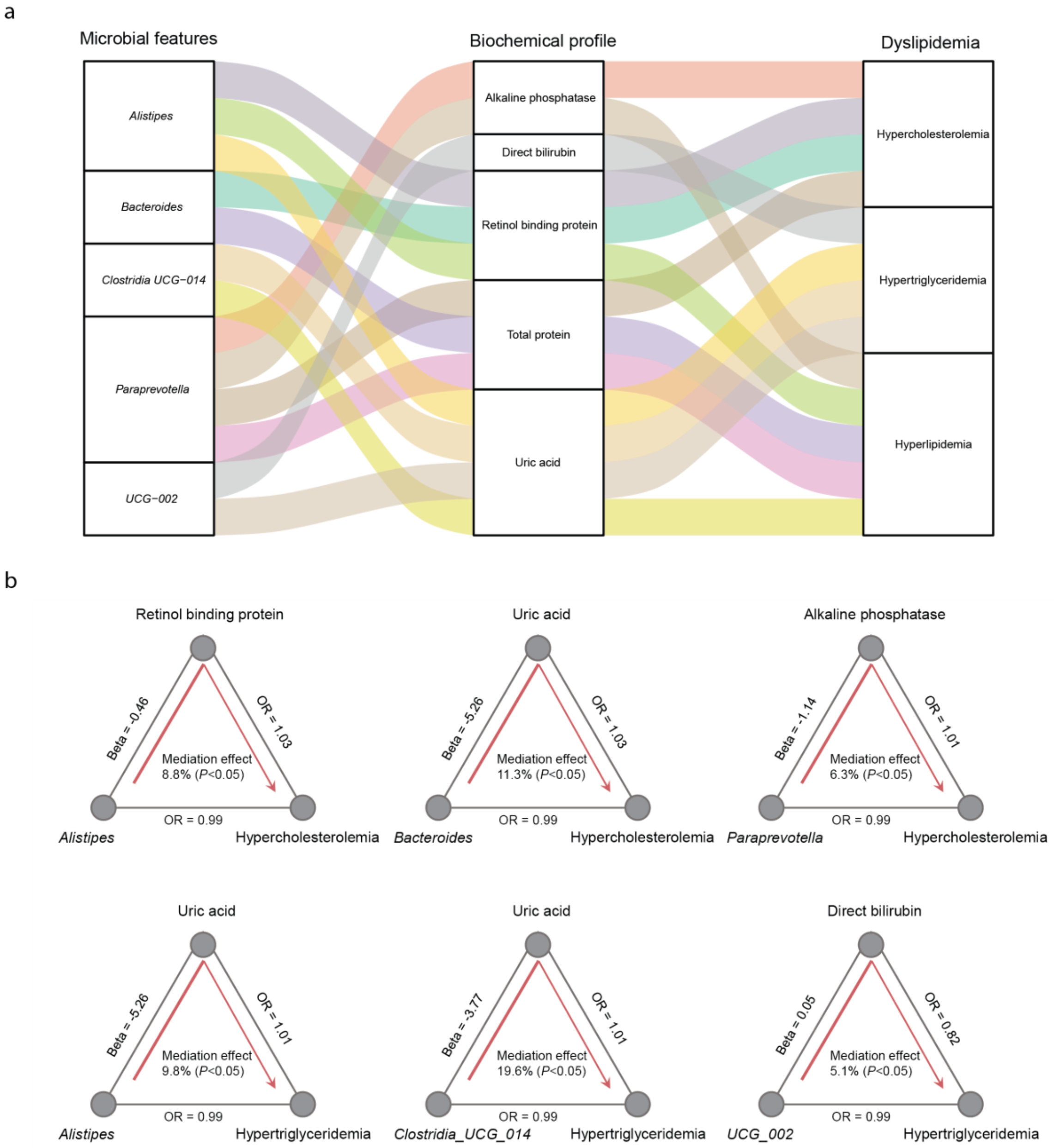

Supplementary Figure 6. Mediation linkages among the gut microbiome, biochemical profiles, and dyslipidemia. a. Sankey diagram illustrating the significant mediation linkages among gut microbiome, biochemical profiles, and dyslipidemia. Columns from left to right show microbial features at genus level, biochemical profiles, and dyslipidemia, respectively. The curved lines across the columns indicate the mediation effects and the colors correspond to different biomarkers; b. Examples of mediation linkages among gut microbiome, biochemical profiles, and dyslipidemia by mediation analysis. The beta coefficient and significance are labeled at each edge, and the proportions of mediation effects are labeled at the center of the ternary diagrams. The red arrows indicate the gut microbial effects on dyslipidemia mediated by blood biochemical parameters.

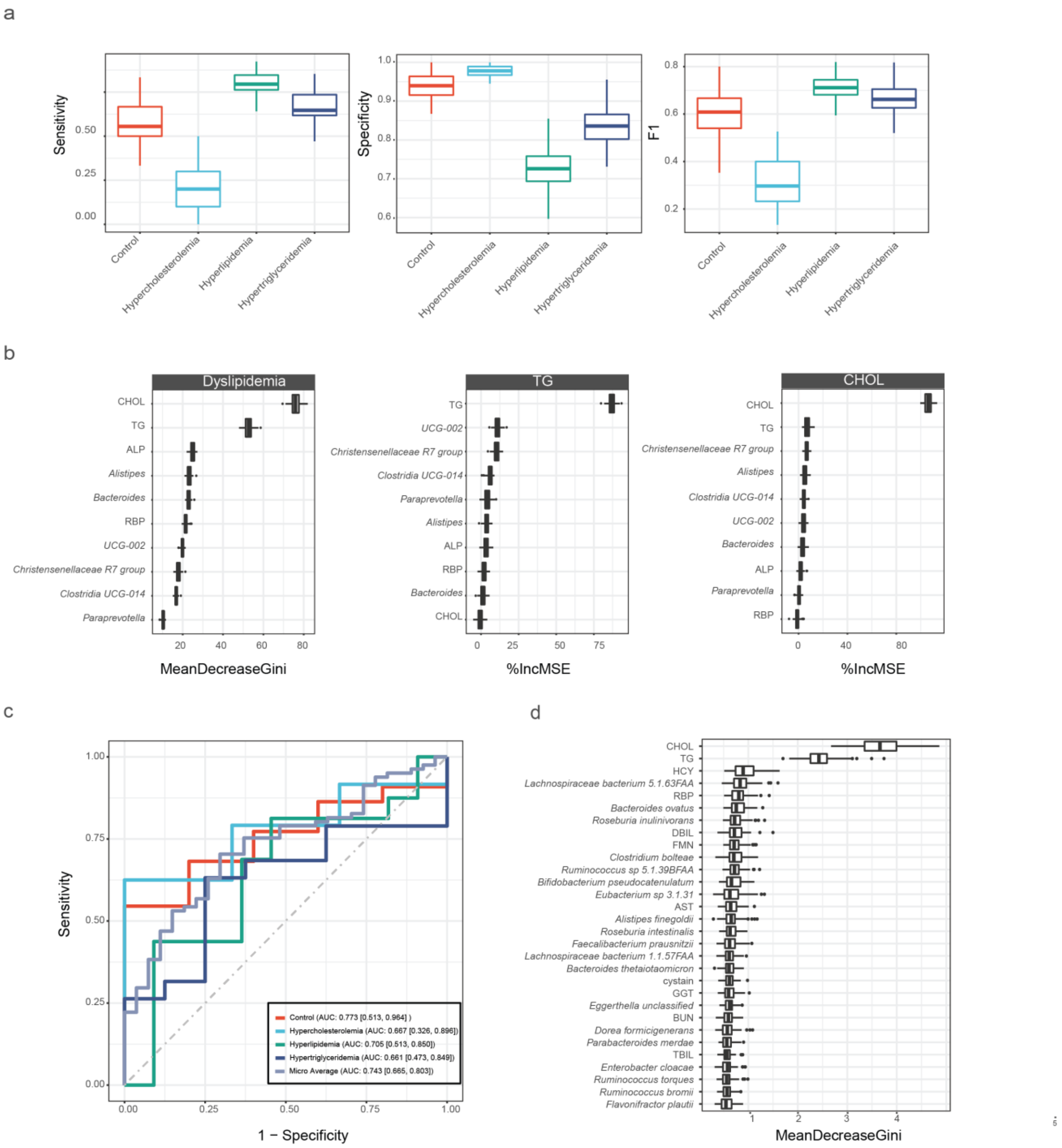

Supplementary Figure 7. Performance of randomforest classifier discriminating dyslipidemia groups and predicting lipid levels. a. Sensitivity, specificity, and F1 score among dyslipidemia groups; b. Importance of selected predictors in classifiers among dyslipidemia groups and lipid levels; c. Prediction performance of randomforest model predicting dyslipidemia using gut microbiome at the species level combined with biochemical data; b. most important taxa (Top 30) in the classification model among dyslipidemia groups. For boxplots, the center line denoted the median. The boxes covered the 25th and 75th percentiles, and the whiskers extended to the most extreme data point, which was no more than 1.5 times the length of the box away from the box. Points outside the whiskers represented outlier samples.
