# Supplementary material for: Ecological change of the gut microbiota during pregnancy and progression to dyslipidemia
Source: NPJ Biofilms Microbiomes. 2023 Apr 3;9:14. doi: 10.1038/s41522-023-00383-7 (PMC10070613; doi:10.1038/s41522-023-00383-7)
Supplement: Supplementary file 2 — Reporting Summary [file 41522_2023_383_MOESM2_ESM.pdf]

nature portfolio

Corresponding author(s): Yankai Xia

Last updated by author(s): Mar 13, 2023

Reporting Summary

Nature Portfolio wishes to improve the reproducibility of the work that we publish. This form provides structure for consistency and transparency in reporting. For further information on Nature Portfolio policies, see our Editorial Policies and the Editorial Policy Checklist.

Statistics

For all statistical analyses, confirm that the following items are present in the figure legend, table legend, main text, or Methods section.

n/a	Confirmed	
☐	☒	The exact sample size (*n*) for each experimental group/condition, given as a discrete number and unit of measurement
☐	☒	A statement on whether measurements were taken from distinct samples or whether the same sample was measured repeatedly
☐	☒	The statistical test(s) used AND whether they are one- or two-sided<br>*Only common tests should be described solely by name; describe more complex techniques in the Methods section.*
☐	☒	A description of all covariates tested
☐	☒	A description of any assumptions or corrections, such as tests of normality and adjustment for multiple comparisons
☐	☒	A full description of the statistical parameters including central tendency (e.g. means) or other basic estimates (e.g. regression coefficient) AND variation (e.g. standard deviation) or associated estimates of uncertainty (e.g. confidence intervals)
☐	☒	For null hypothesis testing, the test statistic (e.g. *F*, *t*, *r*) with confidence intervals, effect sizes, degrees of freedom and *P* value noted<br>*Give P values as exact values whenever suitable.*
☒	☐	For Bayesian analysis, information on the choice of priors and Markov chain Monte Carlo settings
☒	☐	For hierarchical and complex designs, identification of the appropriate level for tests and full reporting of outcomes
☐	☒	Estimates of effect sizes (e.g. Cohen's *d*, Pearson's *r*), indicating how they were calculated

Our web collection on statistics for biologists contains articles on many of the points above.

Software and code

Policy information about availability of computer code

Data collection	Data from questionnaires, clinical visits and laboratory data was entered using comma delimited files and spreadsheets.
Data analysis	QIIME2; KneadData (v0.7.4) ; Bowtie2(version 2.4.2); FastQC toolkit (0.11.9); MetaPhlAn2 (version 2.7.2) ; HUMANn2 (version 0.11.1); DIAMOND (version 0.1.1); ChocoPhlAn pangenome database (version 0.1.1); vegan (v2.5-7) R package; randomForest (v4.6.14)

For manuscripts utilizing custom algorithms or software that are central to the research but not yet described in published literature, software must be made available to editors and reviewers. We strongly encourage code deposition in a community repository (e.g. GitHub). See the Nature Portfolio guidelines for submitting code & software for further information.

Data

Policy information about availability of data

All manuscripts must include a data availability statement. This statement should provide the following information, where applicable:

- Accession codes, unique identifiers, or web links for publicly available datasets
- A description of any restrictions on data availability
- For clinical datasets or third party data, please ensure that the statement adheres to our policy

The 16S rRNA and metagenomic sequencing datasets analyzed in this study has been deposited in the Genome Sequence Archive under the accession number: CRA010129.

Human research participants

Policy information about studies involving human research participants and Sex and Gender in Research.

Reporting on sex and gender	We recruited pregnant women from the affiliated hospital of Nanjing Medical University between 2017 and 2018 in this study and results only applied to females.
Population characteristics	The study encompassed a total of 513 individuals with median age of 29.2 years old (Table S1). About two-thirds of the study population had median education (high school to bachelor's degree, 73.6%) and were primiparous (74.1%). 6.43% of participants (n=33) were overweight or obese before pregnancy
Recruitment	The study was carried out based on Mother and Child Microbiome Cohort (MCMC) Study. Pregnant women were recruited from the affiliated hospital of Nanjing Medical University between 2017 and 2018. A total of 1527 pregnant women were enrolled in the longitudinal sampling.
Ethics oversight	Nanjing Maternity and Child Health Care Hospital

Note that full information on the approval of the study protocol must also be provided in the manuscript.

Field-specific reporting

Please select the one below that is the best fit for your research. If you are not sure, read the appropriate sections before making your selection.

☒ Life sciences ☐ Behavioural & social sciences ☐ Ecological, evolutionary & environmental sciences

For a reference copy of the document with all sections, see nature.com/documents/nr-reporting-summary-flat.pdf

Life sciences study design

All studies must disclose on these points even when the disclosure is negative.

Sample size	The study was carried out based on Mother and Child Microbiome Cohort (MCMC) Study. The study design is presented in Figure 1a. Pregnant women were recruited from the affiliated hospital of Nanjing Medical University between 2017 and 2018. A total of 1527 pregnant women were enrolled in the longitudinal sampling. Stool samples were collected in the second (T2, 24.14 ± 0.95 weeks) and third (T3, 32.11 ± 0.59 weeks) trimesters of pregnancy. Negative control samples (n=10) were also included during the collection, transportation, and DNA extraction of fecal samples.The study encompassed a total of 513 individuals with both time points having fecal samples and lipid profiles.
Data exclusions	Participants were excluded according to the following criteria: (1) previously diagnosed with diabetes mellitus or GDM, thyroids diseases, intrahepatic cholestasis of pregnancy, tumor, eclampsia or HBV; (2) antibiotics usage within 3 months prior to fecal sample collection; (3) multiple pregnancy, and (4) pregnancy with artificial reproductive technology (ART). We also excluded those women without lipid levels and fecal samples missing at T2 or T3 time points during pregnancy.
Replication	We used generalized linear regression, linear mixed model and wilcoxon test to explore the key taxa associated with lipid profile during pregnancy. The results showed reproducibility of association of key taxa and dysipidemia during pregnancy. Randomforest prediction model was built using 100 times cross validation setups and reported results were averaged over validation folds.
Randomization	n/a
Blinding	There was no control or placebo arm therefore binding was not applicable.

Reporting for specific materials, systems and methods

We require information from authors about some types of materials, experimental systems and methods used in many studies. Here, indicate whether each material, system or method listed is relevant to your study. If you are not sure if a list item applies to your research, read the appropriate section before selecting a response.

Materials & experimental systems

n/a	Involved in the study
☒	☐ Antibodies
☒	☐ Eukaryotic cell lines
☒	☐ Palaeontology and archaeology
☒	☐ Animals and other organisms
☒	☐ Clinical data
☒	☐ Dual use research of concern

Methods

n/a	Involved in the study
☒	☐ ChIP-seq
☒	☐ Flow cytometry
☒	☐ MRI-based neuroimaging
